# Dependency Map of Proteins in the Small Ribosomal Subunit

**DOI:** 10.1371/journal.pcbi.0020010

**Published:** 2006-02-17

**Authors:** Kay Hamacher, Joanna Trylska, J. Andrew McCammon

**Affiliations:** 1 Department of Chemistry and Biochemistry, University of California San Diego, La Jolla, California, United States of America; 2 Center for Theoretical Biological Physics, University of California San Diego, La Jolla, California, United States of America; 3 Interdisciplinary Centre for Mathematical and Computational Modelling, Warsaw University, Warsaw, Poland; 4 Howard Hughes Medical Institute and Department of Pharmacology, University of California San Diego, La Jolla, California, United States of America; Peking University, China

## Abstract

The assembly of the ribosome has recently become an interesting target for antibiotics in several bacteria. In this work, we extended an analytical procedure to determine native state fluctuations and contact breaking to investigate the protein stability dependence in the 30S small ribosomal subunit of Thermus thermophilus. We determined the causal influence of the presence and absence of proteins in the 30S complex on the binding free energies of other proteins. The predicted dependencies are in overall agreement with the experimentally determined assembly map for another organism, Escherichia coli. We found that the causal influences result from two distinct mechanisms: one is pure internal energy change, the other originates from the entropy change. We discuss the implications on how to target the ribosomal assembly most effectively by suggesting six proteins as targets for mutations or other hindering of their binding. Our results show that by blocking one out of this set of proteins, the association of other proteins is eventually reduced, thus reducing the translation efficiency even more. We could additionally determine the binding dependency of THX—a peptide not present in the ribosome of E. coli—and suggest its assembly path.

## Synopsis

The ribosome acts as the protein–production facility of the cell. Interfering with its assembly will shut down the function of the cell. The bacterial ribosome differs from the eukaryotic one. Both properties together prompt for the development of antibiotics targeting the bacterial ribosome. To target this macromolecular complex most efficiently, one needs to understand the assembly process. The smaller subunit consists of 21 proteins and a ≈ 1500 nucleotide long RNA chain. This size makes it unfeasible to treat the assembly process with conventional computational techniques. To overcome this size limit, this paper introduces a new approach which computes energetic and entropic contributions to the binding energy of individual proteins. By systematic *Gedankenexperiment*s and an accompanying analysis procedure we were able to deduce the binding dependencies of the proteins and the influences of their respective absence or presence onto other proteins. From the obtained influence map we can deduce potential target proteins for drug development or other binding hindering experiments.

## Introduction

Ribosomes are large ribonucleoprotein assemblies that conduct the process of translation of the genetic code. They are composed of two asymmetric subunits, small and large, which associate through a network of intermolecular interactions. Many antibiotics, which are widely used in the treatment of bacterial infections, interfere with protein synthesis. A large number of them bind to the small ribosomal subunit [1] and block proper ribosome function either by hindering the decoding process or by inhibiting the functional conformational changes of the ribosome [2]. The understanding of interactions governing the assembly mechanism is of great importance because recently it has also been found that the aminoglycoside antibiotics inhibit not only the translation itself but also the formation of the small subunit [3].

**Figure 1:**
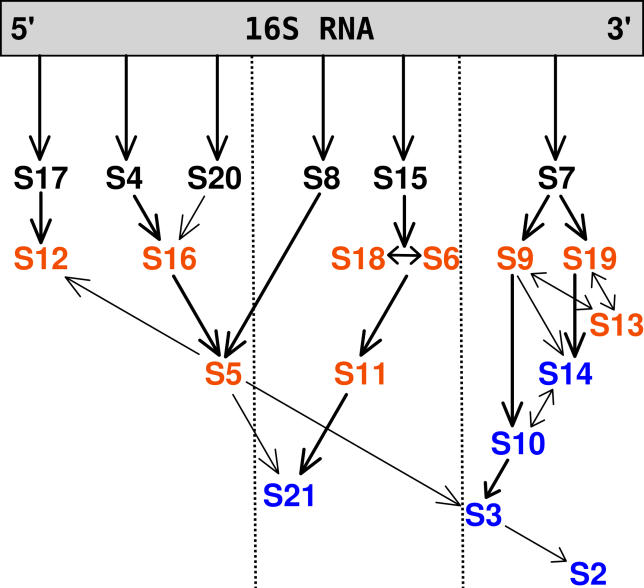
The 30S Subunit In Vitro Experimental Assembly Map for E. coli The primary, secondary and tertiary binding proteins are shown in black, orange, and blue, respectively. Arrows indicate facilitating effect of binding of one protein on another. Adapted from the review of Culver [6] based on the work of the Nomura Laboratory [4,5].

In bacteria, the small and large subunits are named, according to their sedimentation coefficients, 30S and 50S, respectively. The 30S subunit, which is the subject of this study, consists of the 16S ribosomal RNA (16S rRNA), and 21 proteins which are labeled S1, S2, … , S21. During ribosome activity, messenger RNA and transfer RNA molecules bind to the small subunit. The main role of the small subunit is to maintain translation fidelity by assuring for correct decoding. In the early 1970s, it was found that the Escherichia coli small ribosomal subunit can reassemble in vitro from the 16S rRNA and a mixture of the 30S proteins [4,5]. Such reassembly produces an active 30S particle, and these experiments revealed that subunit complexation is a sequential and ordered process. The proteins were classified as primary, secondary, or tertiary binders, depending on their ability to bind alone or only in the presence of other proteins. The experimentally derived assembly order map is presented in Figure 1. Since then, the pathway and the mechanism of the assembly have been of significant interest (for review see [6]), however, many details of this process still remain unclear.

Apart from the “assembly order map” of Nomura and coworkers [4,5], a “kinetic assembly map” was also determined [7]. The kinetics-based map classifies the proteins as early, middle, middle-late, and late binders, and suggests that the assembly of proteins proceeds roughly from the 5′, through central, to the 3′ domain of 16S rRNA even though in vitro this process is not coupled with the temporal order of transcription of the proteins. These two maps (assembly order and assembly kinetics) serve as a model of the ordered assembly of E. coli 30S subunits. Because all the information needed for the small subunit assembly is present in the 16S rRNA and protein components, it should be possible to study this process based on the crystallographic structures of the 30S ribosomal complex.

Apart from the vast amount of experimental approaches to study the association of proteins with 16S rRNA, theoretical modelling approaches of the 30S subunit assembly have not been numerous. Coarse-grained Monte Carlo simulations have been performed to assess the change in fluctuations upon binding of proteins in the 3′ domain assembly [8] and to predict the contributions of each of the proteins to the organization of the binding sites for the sequential proteins in the S7 pathway. Recently, a similar coarse-grained force field was applied in molecular dynamics simulations of the small subunit assembly [9]. However, these two studies focus more on the 16S rRNA conformational changes due to the binding events than on the energetics of the 30S assembly. To account for the latter, we have previously applied an implicit solvent Poisson-Boltzmann model to study the relative binding free energies of 30S proteins to 16S rRNA [10]. The Poisson-Boltzmann all-atom investigation, even though giving encouraging results, was performed on a single 30S subunit configuration and was somewhat sensitive to applied parameters, such as the dielectric constant of the subunit and the placement of the dielectric boundary between the 30S molecule and implicit solvent.

The biggest drawback of all these approaches is that they are time-consuming thus are not applicable to the several thousands of configurations we have made for this study. This huge set of configurations is however necessary to deduce interdependencies in a comprehensive fashion. Therefore, we decided to base this work on a computationally faster but still accurate approach which can focus both on the changes in energetics and in fluctuations. Our model is based on the self-consistent pair contact probability approximation (SCPCP) by Micheletti et al. [11]. The SCPCP has several advantages over other coarse-grained and computationally fast methods: a) while it is based on the fluctuations of residues, it can—in contrast to elastic network models—break contacts between residues, b) the SCPCP free energy also includes entropies that are not accessible by methods that compute binding energies as a sole sum of knowledge based interaction strengths. The latter cannot provide for long-range influences, which we found to be relevant in the assembly (see Results).

We calculated the dependencies of protein binding to 16S rRNA for the Thermus thermophilus small ribosomal subunit and were able to reproduce in many aspects the E. coli assembly map as well as predict the differences of assembly between those two bacteria. We were able to identify key proteins most important in mutual stabilization. In addition, we propose a mechanism of binding for the THX-peptide. In future work, the model may be easily applied to the large ribosomal subunit for which a detailed experimental map of binding is not yet available.

The paper is organized as follows; the Results present the interdependencies of protein binding for the T. thermophilus bacterium derived from computational experiments for the removal from the 30S complex of one or two proteins at a time. The similarities and differences with the E. coli assembly map are discussed. The computational model and the parameterization are presented in the Materials and Methods section.

## Results

### Benchmarking by Comparison to Experiment

The SCPCP allows for the computation of the temperature factors which we compared to B-factors determined in the crystal structure. We used Spearman's ranking coefficient [12] *r_s_* to quantify the agreement, and the results are shown in Table 1. We obtained an overall good agreement except for the S12 protein for which we obtained a significance of 92%. The origin of this deviation remains unclear because we cannot relate this effect to the amount of buried surface area upon binding, protein size, or other quantities that might influence stability. The crystal structure we used [13] was obtained only to the resolution of 3.05 Å. We can, therefore, safely assume that all other computationally derived B-factors are in good agreement with the experiment.

**Table 1:**
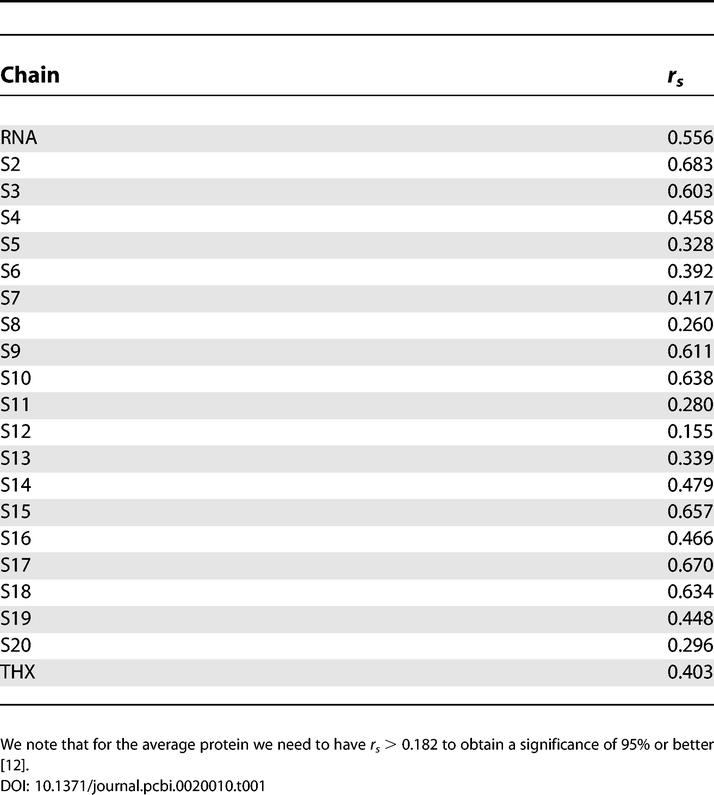
Comparing Computed versus Experimental B-Factors with a Ranking Measure (Spearman's *r_s_*)

Additionally, we compared the computed binding free energies with the available experimental data for T. thermophilus 30S primary binding proteins [14–16]. These experiments determined the apparent dissociation constants for the binding of a single protein to the naked 16S rRNA or its fragments. The binding energies are 269 a.u. (arbitrary units) (51.7 kJ/mole) for S4 [14], 147 a.u. (48.6 kJ/mole) for S8 [15], and 144 a.u. (42.8 kJ/mole) for S7 [16] (with the experimental values given in parentheses). As discussed in the Parametrization section, we do not expect to obtain absolute binding free energies from our computations. We see, however, from the few available experimental data that the SCPCP method is capable of giving the correct ordering of the binding strength in energetic terms.

### Influence Map

#### Influence of removal of one protein from the 30S complex.

First, we performed a computational experiment by removing each of the 20 proteins one at a time from the whole complex. We then computed the binding energies for all the remaining single 19 proteins. From these calculations, we obtained the differences in binding energies for the 19 proteins induced by the absence of every single protein. Therefore, we could quantify the stabilizing effect that the presence of one protein has on the others.

**Figure 2:**
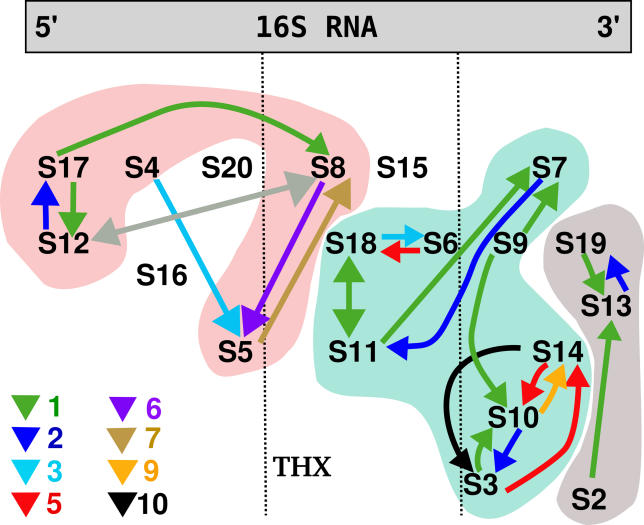
The Influence Network of the Presence of Single Proteins on the Binding Stability of the Other Proteins The three clusters and the four unaffected proteins are visible. The colors of the arrows indicate Δ*_r_* (the larger the number, the stronger the influence). The arrows point from the removed protein to the affected protein, e.g., removal of S4 alters the binding strength of S5 with the “influence strength” of 3. The gray arrows indicate the interaction that is due to very small relative energy change and involves the “suspicious” protein S12 (see text for details).

As confirmed above, the SCPCP gives the correct ordering of binding energies. Therefore, we ranked every removed protein from the remaining proteins according to their binding strengths. The removal of one protein can induce a shift in the ranking, thus indicating the influence of the removed protein on the re-ranked protein. We quantify this influence by the difference in rank Δ*_r_*. The results are shown in Figure 2 and the procedure in a pseudo-language in Protocol S1. The rationale behind such analysis procedure stems from the fact that relative association or dissociation probabilities in the assembly map are governed by the order of binding free energies. If the removal of protein Z changes the relative order from X-more-strongly-bound-than-Y to Y-more-strongly-bound-than-X, then the probabilistic assembly order is also changed, and we attribute an influence of protein Z on protein X.

In our approximation, we find that proteins S15, S16, S20, and the peptide THX are neither influenced by any other protein nor are influencing other proteins. This does not come as a surprise as those proteins are only in contact with 16S rRNA and not with any other proteins. In some cases, we notice, however, subtle correlation effects that can be attributed to a non-local stabilization of proteins. This will be discussed in the next section. All other proteins are found to be the members of three influence clusters. These clusters show a large overlap with the notion of previously obtained assembly maps for E. coli (see Figure 1 for comparison).

The first one of those clusters has eight members (Figure 2, blue shaded area). According to our calculations presented in Figure 2, proteins S6 and S18 influence each other. This is in agreement with the early experimental study of the assembly of E. coli 30S proteins where it was proposed that S6 and S18 bind as a dimer [5,7]. Such dimerization of S6 and S18 was recently proved for a hyperthermophilic bacteria Aquifex aeolicus [17,18]. Also, the influence of S18 and S11 is in excellent agreement with the experimental assembly map of E. coli. The influence of S11 on S7 in Figure 2 is relatively small, and it is not found in the E. coli assembly map. However, in the T. thermophilus structure these proteins are in contact, thus stabilizing each other slightly by the common interface. We have to emphasize that we study the T. thermophilus bacteria, therefore, we expect that our map may differ from that of *E. coli,* and those differences may be interesting to note. In the first cluster, S9 protein facilitates slightly the binding of S10, which is similar to what happens in the E. coli subunit. The influence of S9 on S7 is due to their close proximity in the crystal structure. The strong cluster of S3, S10, and S14 proteins is also present in the E. coli experimental assembly map. All of the three proteins are tertiary binders according to the E. coli map, and it is, therefore, not surprising that their binding may be interdependent. It was proposed that these proteins form a strong hydrophobic cluster [13]. We show that indeed proteins S3, S10, and S14 have a strong influence upon one another.

The second cluster consists of proteins that are bound to the 3′ domain of 16S rRNA (Figure 2, gray shaded area). The mutual relation between S13 and S19 agrees again with the E. coli map and the proposal that these proteins bind together [6]. The influence of protein S2 on S13 is rather weak and can be attributed to a relative energy difference smaller than 0.01%. The same situation holds for S12 and S8 in the third cluster, where the ranking difference is due to a relative energy change of ~0.004% (relative figures with respect to the overall binding energy of the complex). Additionally, we note that the S12 protein was the one whose computed B-factors did not agree well with the experimental ones. Therefore, we have to treat the results obtained for S12 carefully.

The stabilizing mutual effect of S17 and S12 in the third cluster agrees with the E. coli experimental map. The same holds for proteins S5 and S8; in the E. coli map in Figure 1 the binding of S5 is strongly dependent on S8. We also see the impact of S4 on S5 which is present in the E. coli assembly map but it is not a direct influence; instead it involves protein S16. In our simulations of *T. thermophilus,* S16 is not in the influencing clusters. We also see an additional influence of S17 and S12 on S8 which is not suggested for E. coli. This is due to a common interaction surface of those proteins. The interaction of S17 and S8 is further interesting because S8 is binding to the central domain of 16S rRNA and it is therefore believed to be a later binder than S17. If this is the case, then S8 is probably responsible for maintaining the proper fold of the RNA that occurs during protein binding.

By removing one protein at a time and analyzing the effects by means of Δ*_r_,* we were able to deduce a large fraction of the stabilizing influences in the T. thermophilus assembly map. We can, however, proceed further with the procedure of protein removal to investigate the interdependencies in more detail. This is presented in the following section.

#### Second-order stabilization revealed by two-protein removal.

We went one step further in the disassembly by removing every pair of proteins and by computing the binding energies of the remaining ones in the same way as above. Details on the procedure can be found in Protocol S1. The results represent a tensor 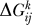 whose values show the induced effect of removing proteins *i* and *j* on the binding free energy of the third protein *k*. We set 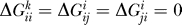 for simplification of notation.

For a fixed *k*, the resulting matrix Δ*G^k^* can be approximated in general by a truncated sum of the form 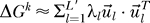 where 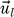 are the *L* = 20 eigenvectors of 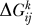 and *λ_l_* are the respective eigenvalues. The eigenvectors and eigenvalues are assumed to be ordered from the smallest to the largest eigenvalue. The upper bound *L′≤L* defines the accuracy of the approximation. This expansion allows us in general to visualize the effects of the removal of two proteins in a very concise fashion by looking mainly at the eigenvectors. The contribution of a protein to the binding free energy is proportional to its respective entry in the eigenvector.

We restricted our analysis to the two eigenvectors that are most important for destabilization. Close inspection of the resulting eigensystems prompts for a modified approximation of Δ*G^k^* as indicated above. We found the distribution of eigenvalues *λ_l_* for all *k* to be dominated by one large negative eigenvalue of magnitude *O*(10^3^), and we encountered 18 positive eigenvalues ~*O*(10^1^) − *O*(10^2^). Note that there is always an additional null-mode with *λ*_2_ = 0 because of our setting 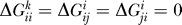 .

**Figure 3:**
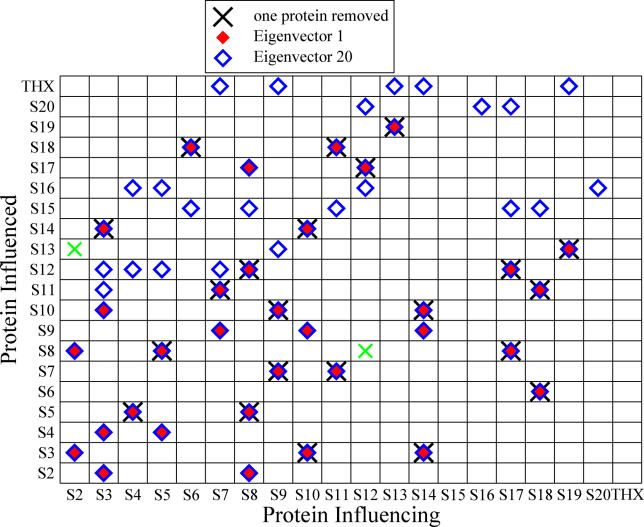
Color-Coded Contribution to Destabilization, Determined by Eigenvector Analysis of the Two-Protein Removal Experiment Red diamonds indicate contributions from the smallest and blue diamonds from the largest eigenvalue, respectively. The symbol × indicates influences that were already found in the one protein removal experiment of the previous section. The green × were discussed in the previous section. The respective energy differences of those two interdependencies are rather small and enter into the magnitude of entries of the eigenvectors only slightly. Entries for 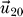 were marked if the value deviated more than 10% from their most likely value, while for 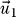 this threshold was set to 1%, reflecting the respective order of magnitude of λ_1/20_.

A destabilization occurs whenever the absolute value of the released binding free energies gets *smaller*. In our sign convention for the *λ_l_,* this is equivalent to either *a small entry in the eigenvector* 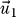 for the eigenvalue *λ*_1_≪0 or a *large entry in* 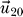 for the eigenvalue *λ*_20_ > 0. The resulting influences are shown in Figure 3. We would like to emphasize that the effects of removing one protein are still present and are found to be in the set of those influences stemming from the smallest eigenvalue *λ*_1_ (Figure 3, crosses).

Close inspection of Figure 3 leads to a new insight beyond the one protein removal experiment presented in the previous section. We can distinguish two groups of destabilizing effects: interactions due to direct contacts (which we will call local) and interactions that occur between proteins that are not in contact (non-local).

First, we present the analysis of the local effects. The removal of protein S2 destabilizes the binding of S8, as they are in contact in the crystallographic structure. Additionally, the removal of S2 weakens its bond to S3 by a local mechanism because both proteins are in proximity, too. In the following contacts, the deletion of the first protein induces additionally a weakening of the second one: S3→S4, S3→S2, S3→S10, S5→S4, S7→S9, S8→S2, S10→S9, S14→S9, S17→S8, and S8→S17. All these influences are local and they overlap with the results from the previous section where we removed only one protein at a time. This indicates the consistency of both our computations and of our analysis procedure.

We also note the following dependencies: S6→S15, S8→S15, S17→→ S15, and S18→S15. As remarked in the previous section, S15 is not in proximity to any other protein. Therefore, its destabilization by all above proteins cannot be caused by the change in the internal energy as this term is local and equal to zero for all non-contacting residues, which is obvious from Equation 1. As we are concerned with the binding free energies, the only other contribution can be entropic. The same argument holds for all the remaining influences. From those subtle contributions, we deduce additional stabilization effects. Moreover, this shows that our procedure also incorporates the non-local effects. Scoring by knowledge-based potentials only could not provide us with such information. In the latter approach, we would observe only those peaks in the eigenvectors that were already found in the one protein removal case, and the effects would be just additive.

**Figure 4:**
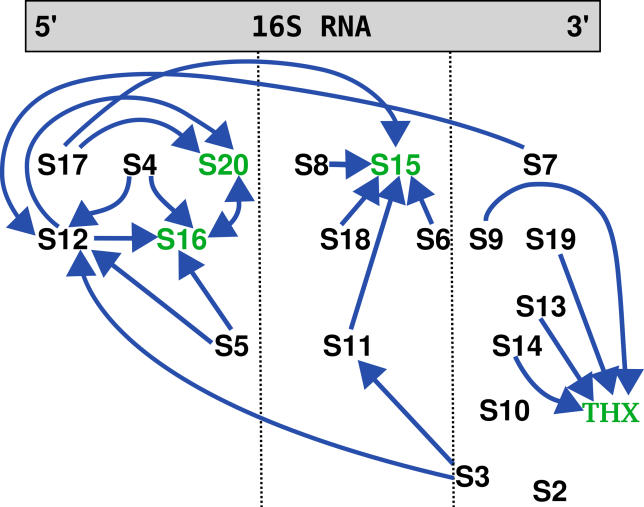
The Additional Destabilization from the Two-Protein Removal Case Mapped onto the E. coli Map (Blue Diamonds in Figure 3) The green proteins are not in contact with any other protein. The peptide THX was placed close to the proteins that influence its binding stability. All interactions are non-local, as the respective proteins are not in proximity.

The entropic non-local effect of (de)stabilization can be explained as follows: consider a protein A that binds to either the whole complex or to a complex that lacks protein B. If B is bound, the RNA is more stiff, thus A encounters a more stiff segment to bind to. In this case, A has to adjust its internal motions and fluctuations to the more rigid binding partner on a greater scale. But reducing the internal motion to a greater amount is accompanied by the release of more entropy, thus Δ*S_B_* > Δ*S*_×_. Therefore, the presence of B stabilizes the bound form of A. It was previously determined [8,9] that binding of the 30S proteins reduces the flexibility of the 16S rRNA and its respective fluctuations, and the above argument holds here, too. This effect is also present in the binding of proteins with local interactions, but the additional contribution from the contact energy makes it difficult to judge which one is the dominating contribution. As the SCPCP takes into account the entropic contributions only from the bond fluctuations, we presumably underestimated the real entropy change. Thus, we expect the effect we described above to be more pronounced in the real molecule. These influences are shown in Figure 4. We note that it is not necessary to compute any entropy. The entropies are naturally deducible from the eigenvalue spectrum, and the fact that every free energy difference between non-contacting residues can only be attributed to entropic effect, as non-contacting residues do not contribute potential energy, as is easily deducible from the Hamiltonian in Equation 1.

#### Assembly as an antibiotic target.

To prevent bacteria growth, one can think of interfering with its protein synthesis through interfering with the assembly of the ribosomal subunit. Recently, antibacterial agents were found to prevent not only the translation process itself but also the assembly of the 30S and 50S ribosomal subunits (see, e.g., [3,19–21]). Aminoglycoside antibiotics, paromomycin and neomycin, were shown to have an inhibitory effect on the assembly of the 30S subunit both from E. coli [3] and from Staphylococcus aureus [19]. Therefore, subunit formation and translation are both targets for antibiotic inhibition. It was also found that the small subunit assembly is hindered by mutations in certain 30S proteins (e.g., S4, S5, S7, and S17 [6], and references therein).

Hence, it seems to be most effective to not only inhibit the binding of just one protein but, instead, of several at the same time. Also, if one prevents an “influential” protein from binding to the 16S rRNA, one not only decreases the association rate of that particular protein and, therefore, translational effectiveness but also hinders the binding of the influenced proteins, making it even more unlikely to obtain a functional subunit. In terms of chemical kinetics, if the absence of a protein *A* increases the dissociation rate *k_off_* of other proteins *B_i_* in its influence cluster by a factor of 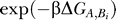 , then the overall equilibrium is shifted away from the functional ribosome to partially formed preproducts by an amount 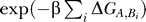 . We assume for simplicity that the *B_i_* are not influencing each other. These higher-order effects would even amplify the destabilizing effect we are suggesting. If we assume all the Δ*G* to be somehow distributed, this factor is in general larger than just the factor induced by reducing the binding of another protein *C* by some other value Δ*G_C_*.

Close inspection of the resulting dependencies presented in Figures 2 and 4 point to S14 or S10, because this would also lead to a smaller amount of bound S3, S10, and THX, or S14 and S3, respectively. Another attractive choice would be either S8 or S5 in the left cluster of Figure 2 or S6 or S18 in the middle group. Such a choice would also destabilize S15. As it is known that the 30S proteins bind roughly from the 5′ to the 3′ domain of 16S rRNA [7], we suspect that the most likely candidates are S14 or S10. These proteins are late binders, and because we study herein the binding of proteins to a perfectly folded structure of 16S rRNA, our simulation setup is closer to an experimental situation for late binders than for early ones. The early binders may in theory bind to only partially folded 16S rRNA.

#### Predictions for the peptide THX.

For the THX peptide, we found an influence from S9, S13, S14, and S19 proteins through entropic contributions. As this molecule is not present in the E. coli ribosome, we would like to suggest experiments in this direction to confirm our prediction that THX and the other proteins are related in their assembly behavior.

## Discussion

In this study, we applied an analytic procedure to the problem of the stability of proteins in the small subunit of the ribosome of T. thermophilus. In the first step, we determined several dependencies between the various proteins of the macromolecular complex. We found an overall good agreement with the in vitro determined assembly map of E. coli and have shown the differences in comparison with the T. thermophilus structure. In the second step, we developed a procedure to investigate the effects of interdependent removal of proteins on the stability of the remaining ones. We found additional pathways that are in agreement with the experimental E. coli assembly map. In addition, we have shown that in roughly half of the cases the non-local effects were responsible for the influences. As our model provides for the energetic contributions only in a local fashion, we were able to distinguish entropic contributions from the fluctuational restrictions imposed on the 16S rRNA upon binding of proteins. Additionally, we propose a new path in the T. thermophilus assembly map for the THX peptide which is not present in the E. coli 30S subunits.

We note that while the method uses a knowledge-based potential, electrostatic interactions are implicitly taken into account. To what extent, however, remains unknown. We therefore could not deduce all dependencies. Additionally, we utilized the crystal conformations for single proteins from the 30S subunit crystal structure. This is not necessarily a precise approach because the free proteins in solution might undergo a structural change. On the other hand, the range of coarse graining is large so that small deviations should not make a difference.

In future, we plan to apply this method to study the assembly of ribosomal proteins in the large subunit. We believe that our studies may help in the investigation of further antibiotics that target the ribosomal apparatus of bacteria.

## Materials and Methods

### Computational approach.

The self-consistent pair contact probability approximation (SCPCP) by Micheletti et al. [11] was used to compute an approximation of the binding free energy. In this model, we treat the building blocks of both the proteins and rRNA— namely amino acids and nucleotides—as beads on a chain centered around their respective C_α_– and P– positions. The potential energy of the amino acids and nucleotides is approximated by a sum of harmonic interactions along the backbones of proteins, and by terms which assign a harmonic energy to contacts as long as the displacement is smaller than some *R*. The Hamiltonian of the system may be written in the form:

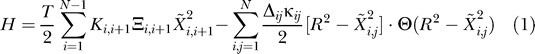
with

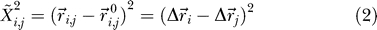



The contact matrix element Δ*_ij_* is 1 if the spatial distance between the heavy atoms of the residues *i* and *j* is smaller than the predefined contact distance which we set to *R_C_*  = 3.75 Å for the heavy atoms (see [22] and the Structure Preparation section for details). 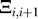 resembles the backbone connectivity and is 1 if *i* and *i* + 1 are covalently bound and 0 otherwise. 
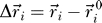
 is the displacement vector of an amino acid or nucleotide from its native conformation 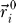 . *K_ij_* is the strength of the pseudo-bond between beads *i* and *j,* while *κ_ij_* assigns a bead-specific contact energy to the beads (see the Parametrization section for details).

One can try to integrate the Hamiltonian of Equation 1 by molecular dynamics simulations [23]. This would be close to simulating the system in a Gō-like fashion [24]. Micheletti et al. [11] proposed, however, a more efficient self-consistent recurrence that allows for both an analytic and a fast treatment.

The contact probability is defined 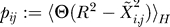 as the thermodynamic expectation value of the contact defining function. We can then replace 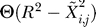 in Equation 1 by *p_ij_*. In this mean-field approximation, we obtain Gaussian-like integrals for the partition function. It is then possible to derive a recurrence relation for the *p_ij_* that converges very fast. The relation reads for the *n* + 1 iteration

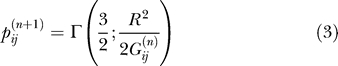
where

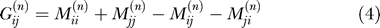
and


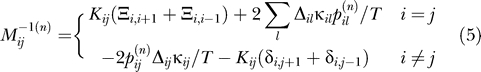


Now we can compute the free energy most efficiently by

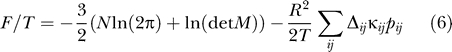



The free energy arises from two different sources: a) the sum of contact energies from knowledge-based potentials, and b) entropies arising from fluctuations. While the last term can in principle be computed from elastic network models or estimated otherwise [25], the SCPCP is a more detailed approach as it allows for the breaking of contacts.

### Parametrization.

For the amino acid–amino acid interactions 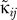 we apply the values from the knowledge-based potentials of Miyazawa and Jernigan [26]. These values were already successfully used with the SCPCP method on a more coarse-grained level in the study of binding of bovine pancreatic phospholipase A2 [27]. The interactions between different proteins were weighted according to the values reported in [28]. The intra-RNA contacts were assigned a strength of 2.51*RT*, the protein–RNA interactions an energy of 2.83*RT* [9,29,30]. For *K_protein_* we set 83.33*RT* as in [30], while for the RNA we set softer bonds with *K_RNA_* = 5*RT* as in [30]. Table 2 and Table 3 show those values in widely used units of kcal/mole.

**Table 2:**
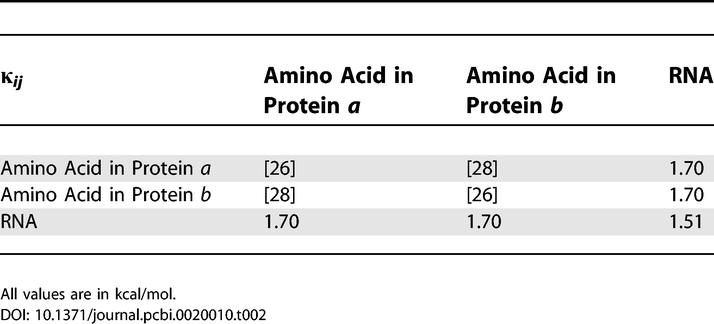
The Contact Interaction Strengths Applied in Our Model

**Table 3:**
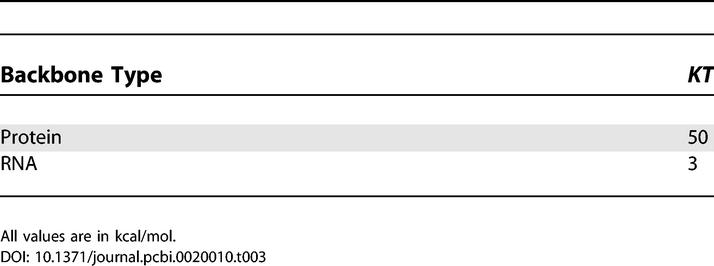
The Covalent Bond Strengths Applied in Our Model

We adjusted the *κ_ij_* for the well depth by setting 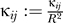 so that the contacts in the native structure retain the Miyazawa-Jernigan contact energies in the Hamiltonian (Equation 1).

Binding energies are now computed as the difference of the free energies between the complex and the binding partners—obtained with three separate SCPCP computations. With this approach, we do not expect to obtain exact binding free energies as, e.g., solvent effects are taken into account only implicitly by the usage of knowledge-based potentials. We have merely chosen the values above to weight the interactions according to their strength. Therefore, we referred to the obtained values in the preceding parts of this paper as being measured in a.u.

Additionally, we would like to emphasize that the results are not sensitive to the choice of the parameters (see below). They were merely chosen to obtain reasonable energy scales. With the suggested method, we cannot reveal mechanisms of molecular recognition, but we can reveal the mutual influence of binding partners in larger macromolecular complexes.

### Sensitivity to parameters.

We tested the stability of the results with respect to our chosen parametrization. To this end we first constructed two different tests: a) we averaged the respective values of Keskin and Miyazawa-Jernigan and assigned to every protein–protein contact an interaction energy *κ_intrachain_* = 3.58RT, and for every internal contact in a protein a value of *κ_intrachain_* = 3.18RT; and b) we assigned to every protein contact—regardless whether internal or external—an overall average of *κ_uniform_* = 3.37RT.

We repeated the two-protein removal tests and obtained the eigenvectors 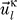 and eigenvalues 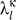 for both sets of *κ*s and for every protein in the same fashion as described above for 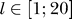 .

Again the eigenvalues λ_1_ and λ_20_ showed the same behavior, so we proceeded with a sensitivity analysis of the eigenvectors which reflect the influence of proteins on each other as given by the respective vector entries. For this analysis, we computed the angle 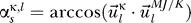 between the obtained eigenvectors in the test and the “full” computation (using the Miyazawa-Jernigan/Keskin interaction values) from above. *κ* reflects the two test sets and 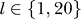 . The *s* indicates that this angle is a sensitivity parameter: if the angle is small, the eigenvectors agree very well and the predicted influences are the same.

For the eigenvector number 1, we found every angle to be smaller than 3°. We averaged the angles over all the proteins subject to any influence and obtained 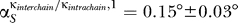 and 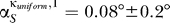 , respectively.

**Figure 5:**
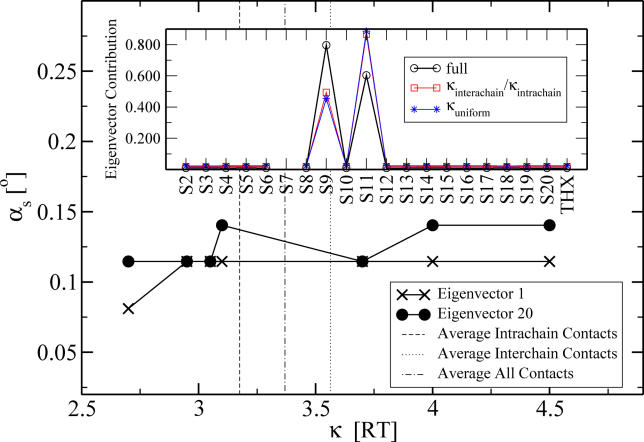
The Angle α*_s_* between the Respective Eigenvectors for an Interaction Strength *κ* of Protein S12 and for the Full Parameter Set The broken vertical lines indicate the average values used in the first two validation experiments in the section Sensitivity to Parameters. The full parameter set refers to the one in the Results section under Influence Map, Second-order stabilization revealed by two-protein removal. Inset: Illustration of the deviation in the 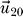 eigenvector contributions for the two different average interaction values (shown for the worst case of S7).

For eigenvector number 20, we found 18 out of the 20 angles to be smaller than 3° for both sets of *κ*. Their averages were 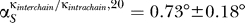 and 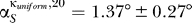 , respectively. The two proteins (S7 and S9) for which the 20th eigenvector showed a larger angle (≈20°) were analyzed further. We plotted the relevant entries for S7, which showed the larger angle at ≈24° (Figure 5, inset). Clearly the differences in the angle stem from the entries for the influence of S9 and S11. Our 10% rule nevertheless does not fail and will still assign an influence to both those proteins. The influence of the changed parameters is too small to decrease the entries to an amount that they would not be assigned as influential anymore.

To investigate the robustness of the method further, we took an orthogonal approach and changed the interactions of all residues of one particular protein (S12) systematically within a reasonable range of possible interaction energies. We kept all the other interactions at their original Miyazawa-Jernigan/Keskin values. We chose S12, as this protein is subject to most one-protein and two-protein removal influences at the same time and should therefore be most sensitive. In addition, this protein was the one whose performance in the B-factor comparison was the worst. We expect this protein to be most influenced by any perturbations in the interactions. We removed all of the other possible two-protein pairs as above and obtained the eigenvectors of the resulting matrix. We show the angle *α_s_* as a function of the S12-interaction strength *κ* in Figure 5. The overall dependence on *κ* is very small and most likely has numerical reasons. The overall offset of 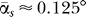 is due to the averaged *κ* in contrast to the distributed *κ_ij_* of the full parameter set of the original calculation. Clearly the relevant eigenvectors 1 and 20 are only subject to a small influence whose impact will be taken care of by the 1%/10% filter rule. As an additional test, we computed *α_s_* also for the protein S12 but changed the interactions of S2 in the same way as above. S2 performed well in the B-factor benchmarking. We found only smallest angles of deviation in both relevant eigenvectors.

In all test cases (averaged and varying *κ*) we found the deviation in the eigenvectors to be very small (roughly smaller than 3°). With this we have shown the robustness of our combination of model and analysis procedure.

### Structure preparation.

The atom positions were taken from a T. thermophilus crystal structure obtained for a 3.05 Å resolution [13] (PDB entry code 1j5e). This particular structure was chosen because of its best available resolution and the fact that it was crystallized as a native 30S complex without any bound ligands. It does not contain the S1 protein, but the omission of S1 does not reduce the ribosome function or prevent the 30S subunit assembly [31]. The structure contains proteins S2 to S20, which correspond to those of E. coli, and a small peptide, THX. The structure of T. thermophilus lacks protein S21.

For the heavy atoms of the 30S structure, we computed the contact matrix 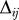 (see Equation 1) with a contact distance of the heavy atoms *R_C_* set to 3.75 Å for both the amino acids and the nucleotides. To account for the stronger interactions in the regions where the RNA forms a double helix and Watson-Crick base pairing takes place, we added some intra-RNA contacts between the phosphate atoms. These contacts were added for only those P···P pairs that could not be found with our choice of *R_C_*. The numbering for the double helical regions was taken from the secondary structure of the 16S rRNA presented in [32]. Additionally, to avoid any long gaps in the structure, we placed the missing phosphate atoms of nucleotides 1535–1538 of 16S rRNA by molecular modelling and visual inspection without adding any further contacts. Therefore, we just introduced a backbone loop for those nucleotides.

### Implementation.

The SCPCP software was implemented using C++, lex and yacc, the GNU Scientific Library, as well as the SuperLU-library for matrix inversion [33]. A BASIC-like configuration language steers the computation. The implementation allows for definitions of abstract sets of contacting entities (e.g., all hydrophobic amino acids in contact with all nucleotides) and their respective strengths.

## Supporting Information

Protocol S1 Details of the One- and Two-Protein–Removal Procedures in a Pseudo Programming Language (85 KB PDF)

## Abbreviations

a.u.: arbitrary units
SCPCP: self-consistent pair contact probability approximation
