# Supplementary material for: Dependency Map of Proteins in the Small Ribosomal Subunit
Source: PLoS Comput Biol. 2006 Feb 17;2(2):e10. doi: 10.1371/journal.pcbi.0020010 (PMC1364506; doi:10.1371/journal.pcbi.0020010)
Supplement: Supplementary file 1 [file pcbi.0020010.sd001.pdf]

Supplementary Protocol S1 for

Dependency Map of Proteins in the Small Ribosomal Subunit

Kay Hamacher[1,2], Joanna Trylska[1,2,3], J. Andrew McCammon[1,2,4]

[1] Department of Chemistry and Biochemistry, University of California at San Diego, 9500 Gilman Drive, La Jolla, CA 92093-0365, USA

[2] Center for Theoretical Biological Physics, University of California at San Diego, 9500 Gilman Drive, La Jolla, CA 92093-0374, USA

[3] Interdisciplinary Centre for Mathematical and Computational Modelling, Warsaw University, Pawińskiego 5a, 02-106 Warsaw, Poland

[4] Howard Hughes Medical Institute and Department of Pharmacology, University of California at San Diego, La Jolla, CA 92093-0365, USA

Correspondence should be addressed to:
Kay Hamacher,
Center for Theoretical Biological Physics and
Department of Chemistry and Biochemistry, MC 0374
University of California at San Diego,
9500 Gilman Drive, La Jolla, CA 92093-0374, USA
e-mail: hamacher_at_ctbp.ucsd.edu
phone +1 (858) 534 - 7256
fax +1 (858) 534 - 7697

1 General Definitions

$S = \{S2, S3, \dots, S20, THX\}$ is the set of all ribosomal proteins. G_M is the free energy as obtained from a SCPCP-calculation for a system containing the rRNA and the proteins of set M.

2 One Protein Removal

The algorithm in a pseudo-programming language reads

```
For each i ∈ S
   Compute ΔG^i_bind = G_S − G_{S\{i}} − G_{i}
Construct a vector of ranks r⃗ according to the respective ΔG^i_bind

For each i ∈ S
   S^(i) = S\{i}
   For each j ∈ S^(i)
     Compute ΔG^{(i),j}_bind = G_{S^(i)} − G_{S^(i)\{j}} − G_{j}
   Construct a vector of ranks r⃗^(i) according to the respective ΔG^{(i),j}_bind

Construct the sets I^(i) of all j that were pushed backwards in
  the ranking by Δ_r^{(i),j} places in comparison to r⃗.
  These sets are the (by i) influenced proteins.
```

The obtained $\Delta_r^{(i),j}$ are the weights on the arrows in figure 2 of the main manuscript. The arrows point from a protein i that (negatively) influences the ranking of a protein j.

3 Two Protein Removal

The algorithm in a pseudo-programming language reads

```
For each i ∈ S
   S^(i) = S\{i}
   For each j ∈ S^(i)
     S^(i,j) = S\{i,j}
     For each k ∈ S^(i,j)
       Compute the tensor element ΔG^k_{i,j} = G_{S^(i,j)} − G_{S^(i,j)\{k}} − G_{k}
       Set ΔG^k_{ii} = ΔG^i_{ij} = ΔG^i_{ji} = 0

For each k ∈ S
   Diagonalize the (20 × 20)-matrix ΔG^k and obtain
    the eigenvalues λ_l and eigenvectors u⃗_l.
   Obtain for l ∈ {1,20} the most probable entry-value U_l in u⃗_l.
   Construct the sets I^k_1 and I^k_20 whose elements are given by the a
    index of the u⃗_l for which |u⃗_{1,a}/U_1| > 1.01 and |u⃗_{20,a}/U_20| > 1.10, respectively.
```

The sets I_1^k and I_{20}^k contain the proteins that influence k. In figure 4 of the main text we again draw arrows from a protein a to the influenced protein k. The set I_{20}^k contains also non-local effects as discussed in the main text.

For the diagonalization procedure we applied the SVD-algorithm in the version described by Press et al. This is necessary - as mentioned in the main text - because of the additional null mode. For a general application we note that the procedure becomes more accurate for larger systems (larger number of chains) as small deviations in ΔG^k (e.g. due to numerics or parametrization) tend to be less relevant the larger the matrices ΔG^k.

Note that the matrices ΔG_{ij}^k are symmetric and one can therefore save a factor of 2 in CPU-time by reducing the loop over j to $j > i$ with $Si > Sj \iff i > j$ and setting $G_{ij} = G_{ji}$ afterwards.
